# Applying speech technologies to assess verbal memory in patients with serious mental illness

**DOI:** 10.1038/s41746-020-0241-7

**Published:** 2020-03-11

**Authors:** Terje B. Holmlund, Chelsea Chandler, Peter W. Foltz, Alex S. Cohen, Jian Cheng, Jared C. Bernstein, Elizabeth P. Rosenfeld, Brita Elvevåg

**Affiliations:** 1 UiT The Arctic University of Norway, Tromsø, Norway; 2 University of Colorado Boulder, Boulder, CO USA; 3 Pearson PLC, London, England; 4 Louisiana State University, Baton Rouge, LA USA; 5 Analytic Measures Inc, Palo Alto, CA USA; 6 Norwegian Centre for eHealth Research, Tromsø, Norway

**Keywords:** Biomarkers, Diagnosis, Language, Human behaviour

## Abstract

Verbal memory deficits are some of the most profound neurocognitive deficits associated with schizophrenia and serious mental illness in general. As yet, their measurement in clinical settings is limited to traditional tests that allow for limited administrations and require substantial resources to deploy and score. Therefore, we developed a digital ambulatory verbal memory test with automated scoring, and repeated self-administration via smart devices. One hundred and four adults participated, comprising 25 patients with serious mental illness and 79 healthy volunteers. The study design was successful with high quality speech recordings produced to 92% of prompts (Patients: 86%, Healthy: 96%). The story recalls were both transcribed and scored by humans, and scores generated using natural language processing on transcriptions were comparable to human ratings (R = 0.83, within the range of human-to-human correlations of R = 0.73–0.89). A fully automated approach that scored transcripts generated by automatic speech recognition produced comparable and accurate scores (R = 0.82), with very high correlation to scores derived from human transcripts (R = 0.99). This study demonstrates the viability of leveraging speech technologies to facilitate the frequent assessment of verbal memory for clinical monitoring purposes in psychiatry.

## Introduction

Our ability to remember stories we have heard can be affected by conditions that affect cortical function. Specifically, the verbal processing component of episodic memory is a useful endophenotype in schizophrenia, with patients displaying a disproportionate impairment in verbal relative to visual episodic memory^1–3^. Indeed, verbal memory assessment is core to virtually every neuropsychological test battery for schizophrenia and for evaluating pharmacological and remediation-based interventions. Unfortunately, verbal episodic memory is traditionally assessed by counting units of information recalled, which requires trained personnel, and limits the tests’ operationalization of what memory actually is (i.e., the ability to recall a certain number of items or themes). Furthermore, only a few test versions exist, which are typically administered in controlled settings (i.e., in the laboratory or clinic) in a cross-sectional manner thus precluding a fine-grained examination on a daily basis of the relationship to clinical state and treatment. In toto this limits scientific progress in terms of applications within psychiatry and its role as a future biomarker or digital phenotype for personalized medicine purposes^4,5^. To address this, we exploited the fact that verbal recall is expressed via speech and that this data stream is potentially suited to processing with modern speech technologies. Our methodology thus moves current assessment practice towards a complete and viable process—from task presentation to automated scoring—by leveraging speech technologies for (i) the administration of the task, (ii) the transcription of voice to text, and (iii) then the application of machine learning logic from previously rated transcripts to produce automated ratings that simulate expert human ratings. This new assessment framework affords a plethora of novel opportunities of clinical value such as frequent monitoring, remote assessment of memory, and most fundamentally enables a detailed examination of the variability in memory at an individual level, which can thus be a critical outcome measure for future clinical trials^6–8^.

We developed a series of verbal memory tests for frequent and self-administrated data collection via smart devices. In the verbal memory task, participants were asked to both immediately—and then after a delay—retell a story that was told to them via the device’s loudspeaker. Ten different stories were developed (e.g., describing what happened at a birthday party) or instructions (e.g., how to assemble a skateboard) such that the stories would be different each day, and that in principle hundreds of stories could be developed to afford a more nuanced and frequent assessment of verbal memory than current tools such as the Wechsler Memory Scale^9^ and Repeatable Battery for the Assessment of Neuropsychological Status^10^. To leverage speech processing technology, the device recorded responses and we derived automated ratings on the text resulting from human transcription as well as automatic speech recognition. We expected automated ratings to correlate well with human ratings. To minimize risk of a usual scenario where machine learning methods are viewed as a mysterious “black box” as a lack of transparency and explainability can make it difficult to understand how an algorithm derived its solution^11^, we sought to keep our rating model simple and interpretable by including only a subset of possible computational features. High correlations between machine scores and human ratings would inspire confidence that employing automated methods can both complement traditional methods^12,13^, and provide a framework in which verbal memory assessment can be a core component of a system for the frequent and longitudinal monitoring of mental states.

## Results

### Administering verbal memory tests using smart devices

**Fig. 1:**
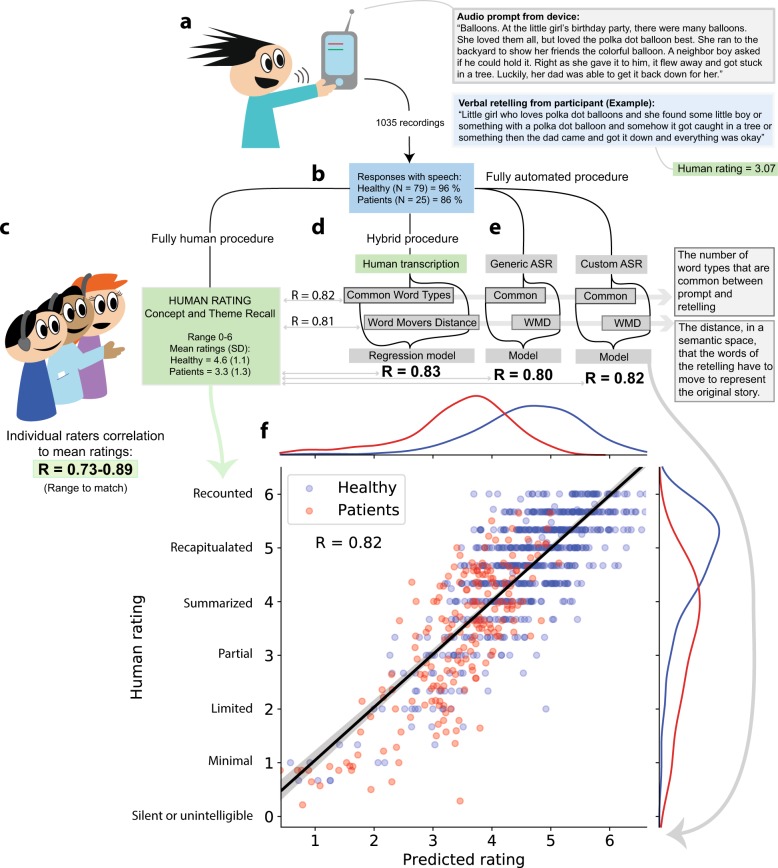
A summary of the procedure for administration and analysis of verbal memory using smart devices. **a** In this example, a story was presented about a girl and her balloons at a birthday party. The participants were asked to “Remember the balloon story, so you can retell it again later”, and both immediate and delayed recall was assessed. **b** A total of 104 participants were tested. Patients tolerated the task but had more trials where they did not provide verbal responses. **c** Humans listened to the responses and rated them for accuracy on a scale between 0 and 6. Our ground truth measure was the average of multiple ratings, and the individual raters correlated with this ground truth between R = 0.73 and R = 0.89. **d** Humans transcribed the response recordings, and the similarity of these transcriptions to the original story was compared. Two features of similarity were extracted, namely a word count procedure and a measure of distance in a semantic space. A regression model produced predicted ratings, and these correlated with average human ratings at R = 0.83, well within the range of individual human raters. **e** The same computational procedure was used on transcripts derived using generic and customized automatic speech recognition systems. The performance of the automated predictive model was still within the level of individual raters with predicted scores correlating with the average human ratings at R = 0.82. **f** A linear model based on transcriptions from the custom ASR system predicted the human ratings well, except for a tendency to assign a higher score to some short responses.

Participants (104 participants, including 25 patients with serious mental illness tested in outpatient care settings; Table 1) were able to easily understand the tasks presented and produced responses and recordings that were of sufficiently high quality such that they were suitable for analysis (Fig. 1, panel A). Ninety two percent of the total of 1035 speech responses were amenable to further processing (86% for patients; Fig. 1, panel B), a critically important finding given that most research on speech has been conducted in controlled laboratory settings. The retellings were on average 61 words (healthy participants’ mean = 62.2 words, SD = 21.4, and patients’ mean = 48.7 words, SD = 22.4; Cohen’s d = −0.8, t = −9.1, *p* < 0.001), with a skew towards more short (<10 words) responses in patients (e.g., “I don’t remember”; healthy = 5.4%, patients 19.7%—Table 2).

**Table 1:** Description of participants and story recall trials.

	Patients (*N* = 25)		Healthy (*N* = 79)	
	M (SD)	Range	M (SD)	Range
Age, years (SD)	49.7 (10.4)	30.0–67.0	21.7 (1.4)	18.0–26.0
Education, years (range)	12.3 (1.4)	7.0–16.0	^a^	12.0–13.0
% female	52.2%		62.0%	
Brief Psychiatric Rating Scale^b^				
Affective	2.1 (1.0)	1.0–5.3		
Agitation	1.6 (0.6)	1.0–3.8		
Positive	2.2 (1.2)	1.0–5.5		
Negative	2.1 (1.0)	1.0–5.5		
Number of story recall trials	354		681	
Responses with recognizable speech (%)^c^	86.0%		95.9%	
Responses < 10 words (%)	19.7%		5.4%	

^a^The exact education level for healthy volunteers was not registered but estimated to be within this range, according to their current academic progress (i.e., they were students attending an undergraduate university course).

^b^Presence of symptoms rated on a 1–7 scale (not present-extremely severe).

^c^Words detected by human transcribers and both ASR systems.

**Table 2:** Description of calculated measures, by group and transcription method.

	Patients (*N* = 25)		Healthy (*N* = 79)		d	*t*	*p*
	Mean	SD	Mean	SD			
Human rating (0–6)	3.3	1.3	4.6	1.1	1.1	13.4	<0.001
Word count	48.7	22.4	65.2	21.4	0.8	9.1	<0.001
Common types, calculated from							
Human transcription	16.4	6.8	26.7	8.1	1.4	17.8	<0.001
Generic ASR	14.4	6.5	25.4	7.9	1.5	19.7	<0.001
Custom ASR	16.5	6.5	26.7	7.9	1.4	18.3	<0.001
Word Mover’s Distance, calculated from							
Human transcription	1.7	0.5	1.3	0.4	−1.0	−12.0	<0.001
Generic ASR	1.8	0.5	1.3	0.4	−1.2	−14.3	<0.001
Custom ASR	1.7	0.4	1.3	0.4	−1.1	−12.6	<0.001
Predicted scores, calculated from							
Human transcription	3.4	0.9	4.6	0.9	1.3	15.4	<0.001
Generic ASR	3.4	0.8	4.6	0.9	1.4	17.8	<0.001
Custom ASR	3.4	0.9	4.6	0.9	1.3	15.8	<0.001

*d* Cohen’s d, *t* Welchs *t*-test, two-sided, *p* *p*-value, Holm-corrected.

### Rating the performance of recall

Human ratings of recall recordings showed the expected pattern where healthy participants received higher scores than patients. This was expected due to numerous differences between the two groups on factors such as illness, age, and education, and dictates that group differences in this study are not be interpreted as specific to memory functions per se. To assess recall performance, we had expert human raters (three to seven raters with higher education in either psychology, cognitive neuroscience or medicine) listen to each recording and rate the recall responses on a 0–6 scale that we had developed to capture the quality of the recall in terms of concepts and themes that were recounted (Fig. 1, panel c). The average rating for recall of concept and theme was 4.3 (SD = 1.3), with higher ratings assigned to responses by healthy participants (Mean = 4.6, SD = 1.1) than by patients (Mean = 3.3, SD = 1.3, Cohen’s d = −1.1, t = −9.1, *p* < 0.001; Table 2). While this study was intended to assess the use of speech technologies over a wide range of performance levels, future studies will be able to make more specific inferences about the role of serious mental illness on verbal memory performance by comparing patients with control participants matched on key variables such as age and levels of education.

On average, each of the individual raters scores correlated with the gold standard rating at R = 0.83 (ranging between R = 0.73–0.89), and it was this level of reliability of rating that we expected an automated procedure to operate within, if it is to be considered sufficiently robust so as to be useful. Among the 21 pairs of raters, the average inter-rater correlations at the response level was 0.73, which supports the notion that the human raters were able to employ the rating scale quite reliably. As is desirable with a task design that seeks to be sensitive to differences, there was a large variance in performance, notably in patients. We conclude that the administration procedure was successful in collecting speech responses that could serve as the basis for assessing verbal memory performance.

### Can automated assessment methods emulate human ratings?

Automated assessment of verbal recall requires both that speech recordings are converted to text, and that there is a method to compare the resulting text to the original story in order to evaluate the amount of details remembered. To examine the viability of these different components, we first examined results generated via a procedure where humans transcribed the recordings (Hybrid procedure; Fig. 1, panel d), before secondly employing generic (‘off-the-shelf’) automatic speech recognition (henceforth ASR), and then finally a customized (‘in-house-developed’) ASR (Fig. 1, panel e).

Simply counting the number of words that were in common between the transcriptions and the original story was highly predictive of human ratings. That higher word counts generally result in higher scores is well documented in other fields (e.g., the automatic grading of essays in education^14^). The correlation between this nonlinguistic surface feature and the average human ratings was R = 0.82. This is a logical finding since the similarity will depend upon the complexity of the materials produced (i.e., the actual recall), and repeating a diverse and complete set of words should correspond to an impression of a good recall performance. Healthy participants produced more common word types (Mean = 26.7, SD = 8.1) compared to patients (Mean = 16.4, SD = 6.8, Cohen’s d = 1.4, *t* = 17.8, *p* < 0.001; see Table 2).

The accuracy of the automated ratings could be further improved by including measures of utterances that were semantically similar to the original words but not identical (e.g., “father” vs. “dad”) using word vector methods. Word vector methods utilize mathematical techniques where a spatial representation of a word meaning is created by analyzing the co-occurrence of words in large language corpora^15–18^. In these so-called meaning-spaces, words that co-occur and have similar meanings are located close to each other, thus allowing for the use of distance as a measure of semantically similarity. The metric Word Mover’s Distance^19^ is suitable for comparing the similarity of the original story to the actual recall because it captures the meaning of words as well as a notion of how semantically distant each word in a text is to its closest aligned word in the other text on which it is to be compared. The Word Mover’s Distance between the recall and the original story correlated with the average human raters (R = −0.81), and healthy participants produced recalls with shorter distances (i.e., more similar) to the original story (Mean = 1.3, SD = 0.4) compared to patients (Mean = 1.7, SD = 0.5, Cohen’s d = −1.0, *t* = −12, *p* < 0.001).

#### Combined feature model

The count of common words and the semantic similarity measurements were combined in an ordinary least squares linear regression model to predict human ratings on par with individual human raters. The weighted model correlated with human ratings at R = 0.83 (range 0.74–0.90 across 5 cross-validation folds), with a regression coefficient of 0.15 for common word types, and −0.54 for Word Mover’s Distance. The combined model accounted for an additional 2% of the variance over just using the simpler measure of common word types, which is not hugely impressive, but the resulting model is more robust against loss of score due to use of words that are not exactly the same as in the original story, but nonetheless have similar meaning (e.g., synonyms). The overall model provides a good fit to the average human ratings, accounting for 69% of the variance and performing at, or just slightly above, the average human raters. Not surprisingly, computed ratings were different between groups, with retellings from healthy participants receiving higher predicted ratings (Mean = 4.6, SD = 0.9) as compared to those from patients (Mean = 3.4, SD = 0.9, Cohen’s d = 1.3, *t* = 15.4, *p* < 0.001). This finding is as expected and simply strengthens the notion that this automated procedure to speech provides valid scores with sufficient variability that can be leveraged in future studies to detect significant cognitive changes within patients across time (i.e., sensitive enough to be used within participants). Indeed, we note that the traditional concern about ‘matching’ groups in a classic clinical sense is both less necessary and more improbable for machine learning studies that specifically can leverage this enormous variability that is inherent in large and ‘messy’ datasets^20^.

To examine the viability of a fully automated system, we used two ASR systems to automatically transcribe speech and compared accuracy with human transcriptions, and found them both to be efficient and accurate. The retelling of specifically constructed and presented stories has the benefit that the participant does not have to reveal any personal or sensitive information, and we had the resources and opportunity to screen the recordings for any unprompted instances of such information, ensuring that sensitive information was not uploaded to the cloud-based ASR system. However, more effective use of cloud-based tools is possible by implementing and maintaining advanced architectures for data management that can be compliant with the strictest legislations, processing data of any level of sensitivity in a safe manner.

**Fig. 2:**
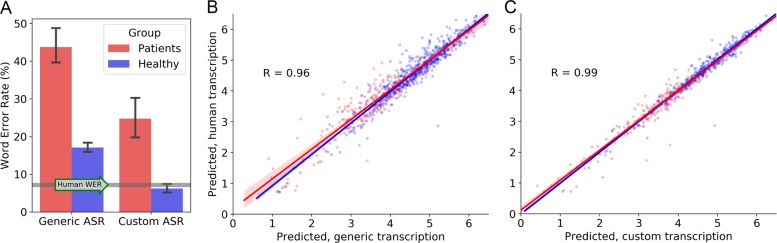
Accuracy of the automatic speech recognition (ASR) systems was different between the two ASR approaches and the two groups, but this did not have a large effect on predicted ratings. **a** The ASR had a lower word error rate on responses from healthy participants compared to responses from patients. The word error rate was also lower on an ASR system customized to the verbal memory task, compared to a generic, off-the-shelf system. The custom system approached the level of errors from human transcribers (7.2%), level indicated by the grey horizontal line. Error bars represent the 95% confidence intervals of the means. **b** Scores from a predictive model using natural language processing methods on human transcription was highly correlated with scores derived using transcriptions from a generic system with higher word error rates. **c** Scores derived from transcriptions using a customized ASR system with lower error rates correlated even better with scores derived using the resource-demanding human transcription procedure, arguably producing equivalent results.

Automatic speech recognition performed using the latest Google’s speech-to-text service produced an overall word error rate of 23.3%, with lower error rates in healthy participants (17.1%) compared to patients (43.7%; see Fig. 2, panel a). This high error rate is likely due to the fact that the Google language model was trained on general language rather than the language specific to our task. Even so, the predictions of a combined feature model based on transcriptions from the generic ASR procedure correlated surprisingly well with human ratings at R = 0.80 (range 0.74–0.88 across five folds). The robustness of such models in the context of high word error rates has been demonstrated in other domains^21^ and is attributable to errors being made mostly on non-essential words, with the arguably more important common type words generally being transcribed correctly.

The word error rate using the customized ASR system was notably lower, with an overall word error rate of 10.5%. In the customized ASR system the language model was specifically tuned towards detecting words that were likely to occur based on our stimulus material (e.g., “balloons” and “skateboards”, not “baboons” and “steakhouse”; for details on equivalent methods, see^22^). Speech from healthy participants was still detected more accurately (6.2% error rate, compared to 24.8% on speech from patients; Fig. 2, panel a). Correlations between computed ratings based on transcriptions from the custom ASR procedure and the average of the human raters remained very high at 0.82 (range 0.74–0.88 across five folds), which was in the range of human to human agreement of 0.73–0.89 (Fig. 1, panel g, shows the predicted ratings vs. the actual human ratings based on the regression model for the automated transcripts). Importantly, the predicted ratings from fully automated procedures correlated highly with results derived using the procedure where humans transcribed the recordings (R = 0.96–0.99; Fig. 2, panels b and c).

In sum, the overall prediction performance derived from transcripts typed by humans (R^2^ = 0.69) vs. those automatically derived using ASR (R^2^ = 0.67) decreased only by 2%, as expressed by variance, a value that in the current assessment context is modest and renders a fully automated system most certainly viable and robust enough to produce data that are clinically useful.

## Discussion

The current study demonstrates the viability and robustness of a method for the frequent and automated testing and scoring of verbal memory that is sufficiently robust that it can be administered outside of controlled settings and where appropriate can be self-administered by the patient themselves. Overall, the procedure was tolerated well by patients and generated high quality speech data. Natural language processing techniques were applied to the speech data and shown to provide novel ways of assessment and scoring of verbal memory. This new framework enables a detailed examination of the stability of memory and its relationship to fluctuations in clinical state within the individual, and as such may generate the critical assays for personalized medicine purposes^4^. Furthermore, this approach offers a practically viable method in ambulatory settings where mobile technology is increasingly used for clinical purposes^23^.

Beyond affording a practical tool, automated semantic techniques can measure more subtle differences in language use that cannot be seen simply in word overlap (e.g., verbatim responses). Although semantic analysis techniques have been shown previously to be effective in measuring the quality of verbal memory by going beyond counting linguistic units and/or themes and accurately measure narrative memory^24–26^, the current study extends previous findings notably by improving upon previously employed methods (Latent Semantic Analysis^15^) by using larger corpora and more modern semantic analysis techniques. This performance improvement is likely due to the fact that newer semantic spaces—such as the one employed in this study—use larger context vectors based on millions of words and incorporate new techniques to measure distance among vectors (e.g., Word Mover’s Distance). From an assessment perspective, this information helps inform that the underlying mechanisms in recall must account for more than rote word recall, and must consider how subtle language transformations, such as recalling the gist^27^, results in accurate recalls.

Combining counts and semantic measures improved on overall prediction and can conceptually be considered a robust baseline of what computational approaches can achieve. Indeed, the 0.83 correlation to the average of the raters of the regression model using the best transcriptions is equal to the 0.83 average correlation of human raters to the average of the other raters. Multivariate models like this can have both performance and utility improved by adding more relevant features, depending on what specific aspects of assessment is important in the context. Intuitively, it may be that additionally weighting the syntax, mostly ignored by the metric of number of common spoken word types, may improve the correlation further. A challenge with developing scoring techniques that depend on word order and syntax is that rules can be non-transferrable to other languages, thus possibly limiting scalability and generalizability of the methods. Expanding the set of features for more clinical relevance, it may be advantageous to assay evidence of language disorganization using speech graph analysis^28,29^ or measures of arousal and emotional valence^30^, using acoustic parameters linked to state-dependent fluctuations in psychiatric symptoms^31^. Although we illustrate the computational natural language processing approach with a test task that is structurally similar to the prose recall subtask (the Logical Memory task) of the Wechsler Memory Scale, the techniques discussed here can likely be applied successfully to other tests with verbal responses, and more broadly to assess if a spoken utterance is relevant to its conversational context.

Transcription of the recalls via automatic speech recognition resulted in higher word error rates as compared to human transcription, but, perhaps surprisingly, the rating prediction model did not show an equivalent decrease in performance with the automated transcriptions. We have previously demonstrated that the same approach can be successfully applied to patients with affective and substance use disorders^32^. This was a group diverse in cognitive ability, with high variability in performance and transcription error rates, but the final verbal recall rating prediction model remained impervious to the non-ideal data. This is promising in terms of reproducibility of the approach in a variety of patient groups. The lack of penalty can partly be explained by the way error rates are calculated, in that variants of words (e.g., “skater”, “skateboarder”) many be counted as errors but not constitute important semantic differences, with such similarities being accounted for by the use of the semantic vector comparisons. Additionally, it may it may be related to the types of errors commonly made by ASR systems, namely errors of inserting, deleting or substituting short and frequently used words like “is”, “in” and “the”, as well as filled pauses such as “uh”, words that will have less consequence when assessing recall performance^33^. Errors may also be specific to certain disorders or accents. Shor et al. found that the five most mistaken phonemes accounted for 20% of errors in a sample of patients with amyotrophic lateral sclerosis, underlining the potential for specialized and tailored speech recognition models for applications in medical settings^34^.

Future studies need to validate the current findings in terms of both the sensitivity to detect changes in memory over time within individuals and establish whether such changes are clinically meaningful at an individual level^35^. Prior work that has employed word vector methods to characterize language in psychiatric patients in terms of semantic coherence has found it useful in predicting differences in patients with schizophrenia and risk of illness onset in psychosis^36–41^, but also that such approaches provide new metrics for analysis of performance in well-established neuropsychological tests^42^. Although our current study does not explicitly establish whether such frequent monitoring is psychometrically viable for ambulatory purposes, we can extrapolate that the current design enables frequent monitoring, and that this specific task can be administered by smart devices both within clinical settings and remotely. Naturally, conducting mental state assessments outside of the controlled setting comes with several practical, technical and legal challenges, which are solvable through interdisciplinary collaboration^43^. For any such implementation to be possible, effective ways to monitor responses by clinical caretakers will be important^44^, both to ensure patient safety in the case of critical situations such as explicit statements of suicidality, and to make sure that the automatic processes are trustworthy and continue to generate fair and accurate scores^20^. Nonetheless, for those patients who have access to digital devices, and can operate such devices with minimal supervision, future assessment methods that embrace mobile technologies promise to be of enormous value in psychiatry and may even enhance the bond between patients and clinicians^45^.

## Methods

### Participants

The participant sample comprised 104 adults. Twenty-five patients were recruited from a group home facility in the Southeastern US (Mean age = 49.7 years; SD = 10.4 years, 52.2% female). The patients all met U.S. federal definitions of serious mental illness (per the Alcohol, Drug Abuse and Mental Health Services Administration Reorganization Act^46^) and they were receiving treatment from a multidisciplinary team. All patients received a standardized clinical assessment battery conducted by trained clinical psychology doctoral students under supervision from a licensed clinical psychologist. This included the Structured Clinical Interview for DSM–IV–TR (SCID)^47^ and the Brief Psychiatric Rating Scale (BPRS)^48^. Information was obtained from the patient, from medical and staff records (when available and access was allowed by the patient) and from staff at the group home. All diagnoses and symptom ratings reflected a consensus by the clinical team, which comprised the aforementioned licensed clinical psychologist and at least two psychology doctoral students who had independently reviewed the case (either in person, or via the video of the interview). Two-thirds of the patients met the criteria for schizophrenia (*N* = 16), and the remaining major depressive disorder (*N* = 8) and bipolar disorder (*N* = 1). The severity of illness in patients was assessed using the BPRS (Table 1). In this scale the severity of self-reported symptoms and observed signs are rated on a scale of 1 (not present) to 7 (extremely severe), and items (e.g., hallucinations, excitement) are combined into “Affective”, “Agitation”, “Positive” and “Negative” symptom categories. The categories were based on a commonly used, empirically-derived factor solution^49^ with some minor modifications to attain acceptable internal consistency. Diagnoses and symptom ratings reflected consensus from the research team. The average scores presented in Table 1 indicated that the sample was relatively asymptomatic overall at the time of testing but there was considerable variability with cases of reported moderate and above severity, represented by cases having category average scores of up to 5.5. Such averages can hide elevations on particular items (e.g., a patient with extreme values within the “Agitations” category may still have an average score of 3.8). The other participants (*N* = 79) were undergraduate students at Louisiana State University presumed to be healthy (henceforth termed ‘healthy participants’; mean age = 21.7 years; SD = 1.4 years, 62% female). The research program was approved by the relevant ethics committee (LSU Institutional Review Board #3618) and all participants provided their informed written consent.

### Procedure and Materials

The recall tasks were developed to run on an iOS software environment—a mobile operating system created and developed by Apple Inc.—and were a part of a larger set of assessment tasks that engaged participants in spoken and touch-based interactions to capture structured daily measures of cognition, motor skills, and language^31,43^. Ten text passages were developed that were to be remembered and retold by the participants. Five of the passages were narrative stories and five of the texts were instructions on how to perform certain actions. The narrative stories were structurally similar to the Logical Memory subtest of the widely used Wechsler Memory Scale^8^ and were between 69 and 87 words in length (average length = 75 words; see Fig. 1 and Supplementary Methods for examples). Each narrative had two characters, a setting, an action that happened in the setting causing a problem, and then a resolution. The instructional passages started with a statement or question about an action that was to be performed, continued with description on how to accomplish the goal of the action, then ended with some concluding details (62 and 83 words in length, average length = 73 words). The passages were presented orally in a male voice and the participant was asked to retell the story immediately with as many details as possible, as well as a second retelling of the same prompt later in the testing session. The mobile device recorded the participant’s retelling.

Every response recording was rated for accuracy on a 0–6 scale by human raters with clinical experience, and the details of the rating rubrics are in the Supplementary Methods. The average of these ratings was treated as the gold standard that the automated modelling approach was designed to predict.

Each recall was independently transcribed by two human transcribers and differences in transcription were resolved, producing an overall human word error rate of 7.2%. Although highly unlikely given the neutral nature of the story recall task, in the rating and transcription procedures the recordings were checked for the presence of directly identifiable information (e.g., names) or sensitive health information. Machine transcription was conducted on the pre-checked recordings using Google’s speech-to-text transcription (https://cloud.google.com/speech-to-text/). However, since such generic tools are built to accommodate speech on a wide variety of topics we also built a customized speech recognizer based on the Kaldi speech recognition toolkit^50^. See Supplementary Methods and refs. ^22,30^ for further details on the transcription procedures.

### Natural Language Processing features for automated rating of passages

When scoring a recall, human raters compare the similarity of the actual recall to the original story that the participant was presented with. In order to create an automated way to score recall it is therefore necessary to develop a model that simulates this process, albeit based upon transcriptions rather than audio per se. Therefore we selected linguistic surface features (e.g., word counts) and semantic content features of the transcribed recall responses to derive a composite score to compare with the human ratings. Prior to analysis, the transcriptions were ‘preprocessed’ (using the built-in string processing methods in the Python programming language; Python Software Foundation, https://www.python.org/) to render suitable for computational methods by for example transforming all text to lowercase, removing punctuation (e.g., commas, periods) and instances of transcribed hesitation markers (e.g., “uh”). Comparison between groups were done with two-sided *t*-tests using the Scipy python module with Holm-correction of p-values using the Statsmodels python module.

First, we computed a simple surface feature describing the similarity between prompt and recall, namely the raw counts of the number of occurrences of particular word types (i.e., individual words only counted once) that were in common between the response and the original prompt.

Second, we computed the distance between the prompt and the recall in a semantic vector space. This means that the words in the original prompt and also the participants’ recall were converted to numerical vector representations that convey the semantic content of the recall. With this method, words are “embedded” in a multidimensional space, where the vectors represent the locating coordinates for words in a way that words with similar meanings are located closer together. These spaces are derived by means of computational language models that are based on analyzing the co-occurrence of words in large language corpora^15–18^. We utilized a set of publicly available word embeddings based on a semantic space with 300 dimensions derived from training a Word2vec model on 240 million words from the Google News corpus^17^. Critically, the semantic similarity between two words vectors may be calculated by measuring “distances” in semantic space between words in the response and their closest related words in the original story presented. Even when the discourse from the prompt and recall have no words in common, based on the embedded word vectors that capture aspects of the semantics, the metrics can assess the “distance” between the two stories (i.e., prompt and recall) in a meaningful way. We calculated this distance between the recall and the original story with the Word Mover’s Distance metric^19^ (using the Gensim software package^51^). Such metrics should produce a measure of the amount of semantic information in common between the recall and the original text.

The two similarity measurements were used as independent variables in an ordinary least squares regression model to estimate the human scores. The correlation between the estimated values and the average human rating was our main performance metric, and to minimize bias in our assessment of model performance we estimated the coefficient using a 5-fold cross-validation procedure. This procedure involves dividing the data into 5 subsets, building the linear model on four of the subsets (i.e., the training sets) while leaving one subset (i.e., the test set) for estimating the correlation coefficient, and repeating this procedure in a fashion such that all subsets had served as both training and test sets. Both the linear models and the cross-validation procedure were implemented using the scikit-learn Python module^52^.

If the predicted scores correlated well with human scores, we may justifiably employ such an automated metric to measure the fidelity of the recall responses with reference to the original story presented. This kind of performance metric would thus be automated, consistent, and objective.

### Reporting summary

Further information on research design is available in the Nature Research Reporting Summary linked to this article.

## Supplementary information

Supplementary Information

Reporting Summary

## Data Availability

The datasets generated during and/or analysed during the current study are available from the corresponding author upon reasonable request and with approval from the LSU IRB.

## Code availability

The code for the similarity measurements, statistical analysis and plotting of Fig. 1f can be made available upon reasonable request.
