# Supplementary material for: Applying speech technologies to assess verbal memory in patients with serious mental illness
Source: NPJ Digit Med. 2020 Mar 11;3:33. doi: 10.1038/s41746-020-0241-7 (PMC7066153; doi:10.1038/s41746-020-0241-7)
Supplement: Supplementary file 1 — Supplementary Information [file 41746_2020_241_MOESM1_ESM.pdf]

Supplementary Methods

Each participant was presented with one narrative and one instructional passage per testing session with the mobile device. The presentation of stimuli items (i.e. the different passages) was counterbalanced using a rotating design such that no one received a repetition of a story across sessions and all stories were sampled across participants.

A narrative story example:

> *"Balloons. At the little girl's birthday party, there were many balloons. She loved them all, but loved the polka dot balloon best. She ran to the backyard to show her friends the colorful balloon. A neighbor boy asked if he could hold it. Right as she gave it to him, it flew away and got stuck in a tree. Luckily, her dad was able to get it back down for her."*

An instructional passage example:

> *"Skateboards. How to put your skateboard together? Set all the parts and your tools out in front of you. Start by attaching the trucks, but don't screw the front one all the way in. The trucks need to be able to move a little so you can turn. After that, attach all four wheels. Turn it right side up and test it out; then make any adjustments you need to on the front truck."*

In the case of the 5 narrative stories, they were additionally prompted to retell the story later on in the testing session (e.g. "*Retell the balloon story again now. Put in all the details you can remember*.") an average of 17 minutes after the original audio appeared and the delayed retelling was done. The participant was given a maximum of one minute to speak, and the time remaining was indicated by a timer bar on devices' screen.

Trained human raters listened to the audio recordings of the recalls and assigned scores as to the quality of the *narrative concepts and recall theme* (i.e., characters, actions, feelings, motivations, names, dates, descriptors, plans, causes, situations). Scores were assigned on a 0 to 6

scale, such that zero represented “silent or unintelligible”, and a high score (6) indicated that all major and almost all minor concepts and/or themes were recalled and that all facts corresponded to the original. All responses were rated by multiple raters (a minimum of 3, maximum of 7) and the average rating was computed.

Raters were given the following instructions:

Please rate the accuracy of the recall of specific themes and concepts from the passage. These can include characters, actions, feelings, motivations, names, dates, descriptors, plans, causes, and situations that were mentioned in the passage. Small transformations in wording on concepts (e.g., "a man" "a gentleman", "a guy") can be counted as equivalent concepts. However, changes in the amount of detail (e.g., "A rainy afternoon", "An afternoon") should be considered as having a different number of concepts.

0 *Silent or unintelligible*.
1 *Minimal*. Few accurate concepts/themes from the original passage, with or without off-topic material.
2 *Limited*. Some accurate concepts/themes, but none of the major concepts/themes. Less than a third of the concepts conveyed.
3 *Partial*. At least half the original material missing; a few major concepts/themes are included.
4 *Summarized*. At least two thirds of the concepts/themes are included and accurate, including two or more important concepts/themes.
5 *Recapitulated*. Most major concepts/themes are included, along with many incidental concepts - accurate concepts.
6 *Recounted*. All major and almost all minor concepts/themes are included. All facts correspond to the original.

Human transcription was conducted via an in-house designed web interface that allowed for audio playback, transcription input and coding of different types of noise and non-speech events and sounds. This careful process allowed us to verify that there were no explicit reference to health information, names, addresses or other possibly sensitive or personal identifying items in the recordings beyond the voice itself. Human transcriptions of speech collected with the mobile application were used to build this custom language model that was particularly designed to detect words and phrases relevant to the audio prompts. The acoustic model used for the custom speech recognition was a Deep Neural Network - Hidden Markov Model [1] trained on all training sets of the Librispeech data.[2] Further details on the speech recognition systems can be found in [3] and [4].

Word error rates for the machine transcriptions were calculated separately for patients and healthy participants (by estimating the minimal edit distance with the Wagner-Fischer algorithm using the “jiwer” software package for Python - https://github.com/jitsi/asr-wer/).

Supplementary References

1. Zhang X, Trmal J, Povey D, Khudanpur S. Improving deep neural network acoustic models using generalized maxout networks. In *2014 IEEE International Conference on Acoustics, Speech and Signal Processing (ICASSP),* Florence, Italy: 2014; 215-219. doi:10.1109/ICASSP.2014.6853589

2. Panayotov V, Chen G, Povey D, Khudanpur S. Librispeech: An ASR corpus based on public domain audio books. *2015 IEEE International Conference on Acoustics, Speech and Signal Processing (ICASSP)* 2015;5206-5210. doi:10.1109/ICASSP.2015.7178964

3. Cheng J. Real-time scoring of an oral reading assessment on mobile devices. In: *Proceedings Interspeech*, Hyderabad, India: 2018;1621-1625. doi:10.21437/Interspeech.2018-34

4. Chandler C, Foltz PW, Cheng J, et al. 2019. Overcoming the bottleneck in traditional assessments of verbal memory: Modeling human ratings and classifying clinical group membership. In *Proceedings of the Sixth Workshop on Computational Linguistics and Clinical Psychology*. Minneapolis, Minnesota, USA: 2019;137–147. URL: https://www.aclweb.org/anthology/W19-3016
